# Supplementary material for: Metformin, Empagliflozin, and Their Combination Modulate Ex-Vivo Macrophage Inflammatory Gene Expression
Source: Int J Mol Sci. 2023 Mar 1;24(5):4785. doi: 10.3390/ijms24054785 (PMC10003317; doi:10.3390/ijms24054785)
Supplement: Supplementary file 1 [file ijms-24-04785-s001.zip › ijms-2115797-supplementary.pdf]

Table S1. RT-qPCR Primers.

Gene	Forward Sequence 5′	Reverse Sequence 3′
Cyclophilin	GGCCGATGACGAGCCC	TGTCTTTGGAACTTTGTCTGCAA
Tnfa	CCAGACCCTCACACTCAGATC	CACTTGGTGGTTTGCTACGAC
Il1b	TGGGCCTCAAAGGAAAGAAT	CAGGCTTGTGCTCTGCTTGT
Ifng	TCAAGTGGCATAGATGTGGAAGA A	TGGCTCTGCAGGATTTTCATG
Il6	CTGCAAGAGACTTCCATCCAGTT	GAAGTAGGGAAGGCCGTGG
Tlr2	CGAATTGCATCACCGGTCAG	CCTCTGAGATTTGACGCTTTGT
Clec7a	CAGAGTGAAGGGCCATGGTT	ACTTGAAACGAGTTGGGGAAGA
